# The complete mitochondrial genome sequencing of *Anisakis simplex* isolated from *Anoplopoma fimbria*

**DOI:** 10.1080/23802359.2019.1673225

**Published:** 2019-10-04

**Authors:** Maolin Wang, Yanqi Gao, Xuejie Li, Wei Wang, Ruijun Li

**Affiliations:** College of Fisheries and Life Science, Dalian Ocean University, Dalian, China

**Keywords:** *Anisakis simplex*, *Anoplopoma fimbria*, mitochondrial genome, full-length sequence

## Abstract

In this study, zoonotic *Anisakis simplex* was isolated and identified from the outermost layer of the stomach of diseased *Anoplopoma fimbria* at an industrial farm in Liaoning, North China (122.1842 E, 39.2616 N). With the completion of *A. simplex* mitochondrial genome sequencing, the full-length mitochondrial genome of *A. simplex* was assembled and analyzed. All results indicate that the complete mitochondrial genome of *A. simplex* was 13,899 bp. There were 20 tRNAs and 12 protein-coding genes (PCGs), and two rRNA all located at the heavy (H) strand. Besides, the phylogenetic tree of 19 *A. simplex* isolated from different host species was constructed. The results showed that *A. simplex* isolated from *A. fimbria* was clustered with *Oncorhynchus nerka* isolate in a clade. To sum up, our research results would further provide essential data for systematics and evolution study of *A. simplex*.

*Anisakis simplex*, belonged to order Ascaridata, family Anisakinae, and genus *Anisakis*, was usually parasitic in the viscera or muscle tissue of much teleost fish. Healthy individuals who eat raw, half-cooked or pickled marine fish that infected by *A. simplex* could appear some gastrointestinal symptoms, such as abdominal pain, nausea, diarrhea and vomiting (Daschner and Pascual 2005). At present, the Chinese government has already listed *A. simplex* as the second-class animal infectious disease pathogen in List of Monitored Infectious Disease in-law.

*Anoplopoma fimbria*, a cold-water economic fish that lived in the deep sea, and belonged to the phyla Actinopterygii, order Scorpaeniformes, family Anoplopomatidae, genus *Anoplopoma*, distributed in the Arctic Ocean, and North Pacific coast, including the North American coast from northern Mexico to the Bering Sea, and the Asian coastal regions from the Kamchatka Peninsula to the northeastern coast of Japan (Rondeau et al. 2013; Xiong et al., 2016). In 2013, the *A. fimbria* was introduced and successfully bred in North China. However, research reports on the disease of *A. fimbria* were still rare.

In the current study, *A. simplex* was isolated and identified from the outermost layer of the stomach of diseased *A. fimbria*, at an industrial farm in Liaoning, North China (122.1842 E, 39.2616 N). With the completion of *A. simplex* mitochondrial genome sequencing by SC Gene Company (Guangzhou, China) via Illumina MiSeq Next-generation sequencing technique, the full-length mitochondrial genome of *H. okamotoi* was assembled and submitted to the GenBank database (No. MK820679). Meanwhile, mitochondrial DNA and specimen of *A. simplex* (Number: AFAS01) were preserved and stored in the Dalian Key Laboratory of Marine Animal Disease Control and Prevention, Dalian Ocean University. All analytical results indicate that the complete mitochondrial genome of *A. simplex* was 13,899 bp. There were 20 tRNAs (tRNA-Lys, tRNA-Ser, tRNA-Ile, tRNA-Arg, tRNA-Gln, tRNA-Phe, tRNA-Leu, tRNA-Thr, tRNA-Cys, tRNA-Met, tRNA-Asp, tRNA-Gly, tRNA-His, tRNA-Ala, tRNA-Pro, tRNA-Val, tRNA-Trp, tRNA-Glu, tRNA-Asn, and tRNA-Tyr), 12 protein-coding genes (PCGs), and two rRNA (all located at the heavy (H) strand. Among these PCGs, nine genes (*nd1*, *atp6*, *nd2*, *cox3*, *nd4*, *cox1*, *cox2*, *nd3*, and *nd6*) were with start codon TTG, the rest PCGs *cytb*, nd5, and *nd4l* used the start codon ATT. Besides, 5 PCGs (*nd1*, *atp6*, *nd2*, *nd4*, *nd6* used the stop codon TAA, and two genes (*nd3* and *cox2*) were with the stop codon TAG.

Based on sequences of 12 PCGs (*nd1*, *atp6*, *nd2*, *cytb*, *cox3*, *nd4*, *cox1*, *cox2*, *nd3*, *nd5*, *nd6*, *nd4l*), the phylogenetic tree of 19 *A. simplex* isolated from different host species was constructed by Maximum Likelihood method. The results showed that *A. simplex* isolated from *A. fimbria* was clustered with *Oncorhynchus nerka* isolate in a clade (Figure 1). Moreover, *A. simplex*, *Anisakis pegreffii,* and *Anisakis berlandi* were clearly divided into different groups (Figure 1).

**Figure 1.**
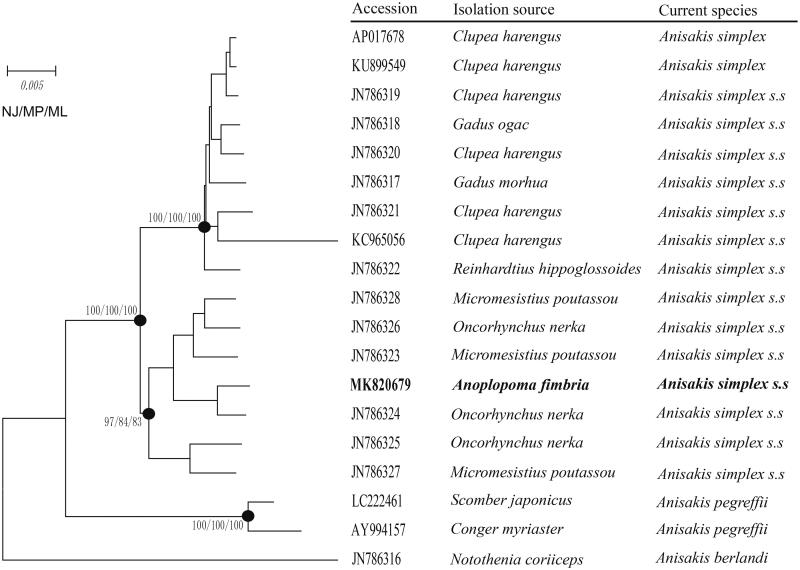
Phylogeny of *Anisakis simplex* phylogenetic tree based on nucleotide sequences of PCGs located in the mitogenome. The number of the branches denoted NJ/MP/ML posterior probabilities.
